# Redo totally endoscopic 3D surgery for recurrent left atrial myxoma causing cerebrovascular accidents with a novel technique: A case report

**DOI:** 10.21542/gcsp.2025.67

**Published:** 2025-12-31

**Authors:** Phan Quang Thuan, Lam Dac Huy, Pham Tran Viet Chuong, Cao Dang Khang, Nguyen Hoang Dinh

**Affiliations:** 1 Department of Cardiovascular and Thoracic Surgery, School of Medicine, University of Medicine and Pharmacy at Ho Chi Minh City, Ho Chi Minh City, Viet Nam; 2 Department of Cardiovascular Surgery, University Medical Center, Ho Chi Minh City, Viet Nam

## Abstract

Background: Recurrent left atrial myxoma is rare but clinically significant due to its potential for embolic complications, including cerebrovascular accidents (CVA). Minimally invasive redo procedures remain technically challenging, particularly in the presence of postoperative adhesions.

Case presentation: We report the case of a 39-year-old Asian female with a history of totally endoscopic myxoma resection via axillary thoracotomy in June 2023, who presented with new-onset right upper limb weakness. Imaging revealed a recurrent left atrial myxoma (20 × 30 mm) attached to the interatrial septum, along with acute ischemic infarcts in the left cerebral hemisphere. The patient underwent successful redo totally endoscopic 3D surgery using a right atrial approach and a double-port (Double-port approach, Camera and main port in the same intercostal space, Totally endoscopic with a skin incision <3 cm, DCT) technique. Partial resection of the interatrial septum was performed to minimize recurrence risk. Cardiopulmonary bypass and aortic cross-clamp times were 110 and 90 min, respectively. Recovery was uneventful, with complete neurological and cardiac function restored.

Conclusion: This case demonstrates that redo totally endoscopic 3D resection of recurrent left atrial myxoma is feasible and safe, even in the setting of pleural adhesions. The right atrial approach, combined with the DCT technique, offers a precise and minimally invasive strategy for managing recurrent cardiac tumors with embolic potential.

## Introduction

Recurrent left atrial myxoma is a rare but clinically significant challenge in cardiac surgery. While myxomas are the most common primary cardiac tumors, recurrence occurs in approximately 2–5% of sporadic cases and up to 22% of familial cases, particularly those associated with Carney complex. Recurrence is often attributed to incomplete initial resection, biologically aggressive tumor behavior, or residual tumor fragments at the site of origin, such as the interatrial septum^1^. Recurrent myxomas can cause severe complications, with CVA being among the most devastating. Embolization of tumor fragments or associated thrombi is the primary mechanism leading to strokes, which may recur multiple times before diagnosis, as evidenced in reported cases. Early recognition and prompt surgical intervention are critical in preventing further neurologic damage.

Redo surgery for recurrent myxomas poses distinct challenges compared to primary resections. Adhesions, distorted anatomy from prior surgery, and the risk of embolization during tumor manipulation demand advanced surgical expertise. While traditional median sternotomy remains the standard approach, minimally invasive techniques, including totally endoscopic 3D surgery, offer unique advantages, such as enhanced visualization, reduced invasiveness, and faster recovery^2^. These approaches are particularly beneficial for patients who have suffered neurologic deficits, enabling early mobilization and rehabilitation^3^.

This report presents a rare case of a recurrent left atrial myxoma causing CVA in a patient who previously underwent endoscopic resection. The case highlights the successful application of redo totally endoscopic 3D surgery to address the recurrence while minimizing surgical trauma and addressing the neurologic sequelae.

## Case presentation

The patient had previously undergone surgical resection of a primary left atrial myxoma in June 2023. The initial procedure was performed through a right mini-thoracotomy measuring approximately five cm along the anterior axillary line (Figure 2A), using direct vision without endoscopic assistance. Cardiopulmonary bypass was established via open right femoral arterial and venous cannulation. After entering the thoracic cavity, the surgical team visualized the cardiac structures directly through the mini-thoracotomy incision. The left atrium was accessed directly via an incision along the interatrial groove, avoiding a right atrial approach and without opening the interatrial septum.

Through this exposure, the surgical team identified a large pedunculated myxoma measuring approximately 5 × 6 cm, attached to the interatrial septum by a distinct stalk. The tumor was excised en bloc, with complete removal of the stalk at its base while preserving the structural integrity of the interatrial septum. Patch reconstruction was not required as the septum remained intact following resection.

The postoperative course was uneventful, and histopathological examination confirmed a left atrial myxoma. Genetic testing for Carney complex was not performed because the patient exhibited no clinical features suggestive of the syndrome, including the absence of lentigines, endocrine abnormalities, or a family history of cardiac tumors.

During the current admission, 18 months after the initial surgery, the patient presented with new-onset right upper limb weakness, which had developed three days prior to hospital presentation. Upon neurological examination at admission, the patient was alert and oriented, with intact cranial nerves and normal sensation. Motor testing showed right upper limb weakness graded 4/5, while all other limbs demonstrated normal strength (5/5). Reflexes were symmetric, plantar responses were flexor, and no aphasia or visual deficits were noted.

**Figure 1.**
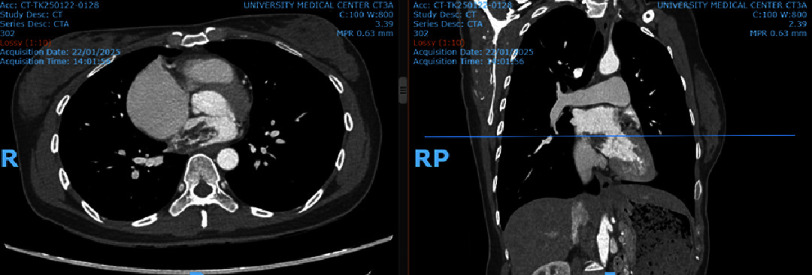
CT imaging demonstrated a left atrial myxoma measuring (20 ×30 mm), attached to the interatrial septum.

Echocardiography revealed a recurrent left atrial myxoma measuring 20 × 30 mm, pedunculated and attached to the interatrial septum, without causing mitral valve stenosis or regurgitation. Computed tomography confirmed the tumor size (Figure 1), and brain MRI revealed multiple small acute ischemic lesions scattered in the subcortical white matter of the right frontal, temporal, and parietal lobes, showing high signal intensity on T2W/FLAIR and restricted diffusion on ADC/DWI.

Several chronic lesions were also noted in the right frontal–parietal regions with low signal on GRE, suggesting prior microhemorrhages. Additionally, a small non-enhancing lesion in the right cerebellar peduncle demonstrated characteristics consistent with an old infarct. No large-vessel stenosis or occlusion was identified on MRA. To prevent further thromboembolic events, the patient was urgently scheduled for Redo Totally Endoscopic 3D Surgery to remove the recurrent myxoma.

The patient underwent Redo Totally Endoscopic 3D Surgery for resection of a recurrent left atrial myxoma. Under general anesthesia with double-lumen endotracheal intubation, intraoperative monitoring included electrocardiography, invasive arterial blood pressure measurement, near-infrared spectroscopy, and transesophageal echocardiography (TEE). Femoral venous and arterial cannulation was performed percutaneously using the Seldinger technique under TEE guidance to establish cardiopulmonary bypass (CPB).

**Figure 2.**
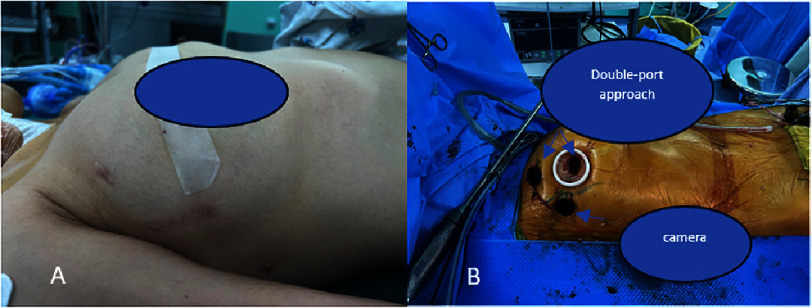
A: previous surgical scar; B: DCT technique approach.

The surgical approach utilized a right anterior mini-thoracotomy through a 2-cm incision at the fourth intercostal space (ICS) near the nipple line, complemented by two accessory ports for instrumentation and visualization. A 10-mm trocar for the 3D camera was placed in the fourth ICS along the anterior axillary line, while a 5-mm trocar was inserted in the second ICS near the mid-clavicular line to accommodate working instruments and facilitate aortic cross-clamping. This configuration constituted the DCT approach (Double-port approach, Camera and main port in the same intercostal space, Totally endoscopic with a skin incision <3 cm) (Figure 2)^4^.

Standard endoscopic cardiac instruments were used through the working and auxiliary ports. The main 2-cm working port accommodated the Aesculap^®^ endoscopic needle holder, endoscopic scissors, and dissecting forceps for tumor excision and septal reconstruction. The 10-mm camera port housed the 3D endoscope, while the secondary 5-mm port was used for Aesculap^®^ endoscopic graspers and suction devices to facilitate exposure, adhesiolysis, and lesion removal.

Moderate pleural adhesions were encountered upon entering the chest cavity, particularly along the inferior and posterior aspects of the right hemithorax. Adhesiolysis was performed using a combination of low-power electrocautery (30 W) and blunt dissection.

**Figure 3.**
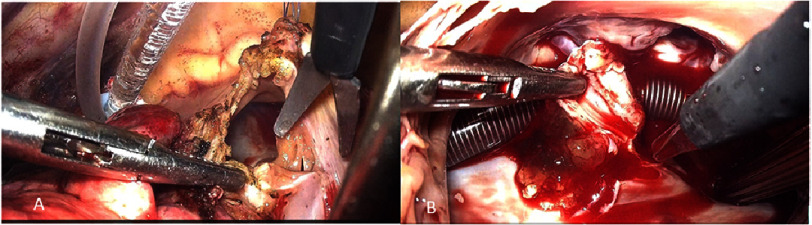
A: right atrial approach; B: partial resection of the interatrial septum and complete excision of the myxoma.

Under single-lung ventilation, a small gauze pad was used to gently depress the right lung to expose the adhesions, allowing precise cautery of the fibrous bands. Sequential lysis was carried out until the pleural cavity was completely freed, ensuring full mobilization of the right lung and safe access to the pericardium. All adhesions were fully released before initiating cardiopulmonary bypass. The patient was placed on cardioplegic arrest, and the recurrent myxoma, pedunculated and attached to the interatrial septum, was excised along with a wide margin of the septum (Figure 3).

A wide resection of the interatrial septal attachment was performed to minimize the risk of recurrence. The excised septal segment measured approximately 3 ×4 cm, encompassing the entire stalk insertion and surrounding tissue margins. Septal reconstruction was completed using a 4 × 6 cm XenoSure^®^ biologic pericardial patch (LeMaitre Vascular, USA), tailored to fit the defect and secured with a continuous 4-0 polypropylene suture, and the heart was restored to sinus rhythm upon weaning from CPB.B

The right atrial approach was chosen because adhesions along the interatrial groove made left atrial exposure challenging and time-consuming, whereas the right atrium was free of significant adhesions, allowing quicker and safer access to the interatrial septum. No intraoperative complications were encountered during the procedure. Tumor excision, septal reconstruction, and cardiac de-airing were completed uneventfully. The patient was weaned from cardiopulmonary bypass smoothly without the need for vasoactive or inotropic support, and the heart resumed sinus rhythm spontaneously.

Total CPB and aortic cross-clamp times were 110 and 90 min, respectively. Two drainage tubes were placed for fluid management. The patient was extubated after three hours and transferred from the ICU within 10 h. Recovery was uneventful, with full muscle strength (grade 5/5) returned before discharge. Transthoracic echocardiography showed normal cardiac function with no residual tumor.

The patient was discharged on postoperative day 2 without complications. Postoperative pathology confirmed myxoma. The patient was not administered any anticoagulant or antiplatelet therapy preoperatively because she was scheduled for semi-urgent surgery.

Postoperatively, low-dose aspirin 81 mg daily was initiated for thromboembolic prophylaxis once hemostasis was secured. At discharge, the patient was neurologically stable. She was alert and fully oriented, with intact cranial nerve function and no speech abnormalities.

Motor examination showed complete recovery of right upper limb strength (MRC 5/5) with normal tone, coordination, and fine motor control. Sensory function was normal in all extremities, deep tendon reflexes were symmetric, and plantar responses remained flexor bilaterally. No residual visual, cognitive, or gait disturbances were observed.

At the 3-month and 6-month follow-up visits, the patient demonstrated completely normal neurological function without residual weakness, sensory deficits, or cognitive impairment. Transthoracic echocardiography performed at 2 months and again at 6 months showed normal cardiac function with no evidence of myxoma recurrence or residual masses. The patient had returned to full daily activities without limitations.

## Discussion

Redo surgery for recurrent cardiac myxoma remains an uncommon clinical scenario, but the timing of recurrence is highly variable and often unpredictable, ranging from a few months to more than a decade after the initial operation. As shown in Table 1, published series consistently demonstrate excellent surgical outcomes, with low operative mortality and acceptable complication rates across various approaches, including sternotomy, thoracotomy, and minimally invasive techniques. Despite these favorable results, totally endoscopic redo surgery is rarely reported in the literature, and most documented cases continue to rely on conventional open or hybrid exposures. This underscores both the technical challenges associated with reoperative minimally invasive surgery and the limited global experience with fully endoscopic strategies in the redo setting.

**Table 1:** Key studies on myxoma recurrence surgery.

**Study (Author, Year)**	**Site of 2nd recurrence**	**Surgical approach**	**Patients (n)**	**CPB time (min)**	**X-clamp time (min)**	**Perioperative complications**	**Recurrence rate**	**Follow-up**
**Bjessmo & Ivert (1997)** ^5^	88% left atrium	Mainly median sternotomy; right thoracotomy (1 case), left thoracotomy (5 cases)	1 recurrence case/63	–	–	Early mortality 1/63; perioperative embolization only in patients without preoperative diagnosis; late complications: atrial fibrillation 7%, thromboembolism 5%	1.6%	Median 13 years
**Keeling et al. (2002)** ^6^	87.7% left atrium	Median sternotomy (47/49); right or left thoracotomy (2 cases)	2 recurrence cases/49	–	–	Early complications in 42.9% (cardiac arrhythmias 22.5%, pericardial effusion 4.1%, respiratory complications 2%, neurologic events 6%, wound issues 8.2%)	2.0%	Up to 24 years, mean 77 months; actuarial freedom from recurrence 95%
**Hermans et al. (2003)** ^7^	Left atrium	Sternotomy (initial and first recurrence); patch closure on first LA resection	1	–	–	None	–	14 years
**Rathore et al. (2008)** ^8^	Left atrium and right ventricle	Right anterolateral mini-thoracotomy with thoracoscopic assistance; beating-heart approach	1	–	–	None	–	12 months
**Mahavar et al. (2021)** ^9^	Left ventricular outflow tract	Redo–redo sternotomy	1	–	–	None	–	6 months
**Bireta et al. (2011)** ^10^	Not specified	Multiple redo sternotomies	1 (Carney complex)	–	–	None	–	13 years

The right atrial approach was chosen for interatrial septal access due to its advantages in cardiac redo surgery^11^. This approach enables complete excision of the myxoma and its stalk, allowing for a wider septal resection to reduce recurrence risk. In redo cases, postoperative adhesions, especially after axillary thoracotomy, commonly affect the interatrial groove, making left atrial exposure more challenging. By avoiding extensive adhesiolysis in this region, the right atrial approach simplifies dissection, reduces surgical complexity, and minimizes the risk of adjacent structure injury, enhancing the feasibility of totally endoscopic redo procedures.

Pleural adhesions in redo cardiac surgery are not an absolute contraindication to a totally endoscopic approach. Notably, adhesions following axillary thoracotomy predominantly affect the interatrial groove, rather than the right atrium, which supports the feasibility of a right atrial approach as an effective alternative. This approach minimizes the need for extensive adhesiolysis, thereby reducing surgical complexity and the risk of lung injury, while also enhancing procedural precision and maintaining optimal visualization in a minimally invasive setting.

The 3D visualization system and DCT approach have been established as standard techniques at our center for totally endoscopic cardiac surgery. This approach, which utilizes only two working ports, offers superior surgical maneuverability, optimizing instrument handling while preserving excellent visualization and precise access to target structures, thereby enhancing the overall efficiency and safety of minimally invasive procedures. Importantly, these principles are universally applied across all access routes—regardless of the specific port configuration—forming the core framework that supports the safety, reproducibility, and efficiency of our minimally invasive procedures. At our center, the totally endoscopic approach is the default strategy for atrial tumor surgery, including redo cases, provided that peripheral cardiopulmonary bypass can be safely established. Patients are considered suitable when femoral cannulation is feasible and there is no evidence of severe systemic atherosclerosis or diffuse peripheral vascular disease that would compromise extracorporeal flow. Only patients in whom peripheral CPB cannot be established due to advanced vascular pathology are excluded and managed with conventional sternotomy.

The literature suggests that recurrence of cardiac myxoma is driven less by the choice of surgical access than by the adequacy of tumor eradication and appropriate risk stratification. Large series have shown that wide excision of the stalk and its surrounding septal or atrial attachment, followed by patch reconstruction, is associated with very low recurrence rates of approximately 1–2%, whereas incomplete resection at the tumor base has been implicated in the few documented recurrences^5^. Recurrent and multifocal cases further emphasize the need for systematic inspection of all cardiac chambers, en-bloc tumor removal without fragmentation, and, in selected high-risk situations, adjunctive measures such as cryoablation of the tumor bed to eradicate microscopic foci^8^. Because familial and Carney-complex–related myxomas carry substantially higher recurrence rates than sporadic tumors, careful clinical and, when available, genetic screening is recommended, together with tailored echocardiographic surveillance—often at 6- to 12-month intervals and continued lifelong^10,12^. To further reduce the risk of recurrence, a partial resection of the interatrial septum was performed, ensuring complete excision of the tumor’s attachment site. This technique is essential for eliminating residual tumor cells, a known factor in myxoma recurrence, and has been widely recommended to enhance long-term surgical outcomes^13^.

The CPB and aortic cross-clamp times in this case were within the expected range for a redo procedure, affirming the technical feasibility and safety of totally endoscopic myxoma resection^1,6^. These findings support the viability of a minimally invasive approach in carefully selected redo cases, offering comparable surgical precision and favorable clinical outcomes while reducing the morbidity associated with conventional open techniques.

Neurological events are among the most feared complications of cardiac myxoma, and embolic stroke has been reported in approximately 30–40% of patients in some series, particularly when the tumor is mobile and friable. Although severe or irreversible deficits are well described, several reports show that early tumor removal can be associated with substantial or even complete neurological recovery, especially in younger patients or when infarcts are small and non-hemorrhagic. In our case, despite the occurrence of an acute ischemic stroke, the patient experienced full restitution of neurological function at follow-up, underscoring the potential for favorable neurological outcomes when prompt diagnosis, timely surgical intervention, and structured postoperative rehabilitation are combined^14,15^.

## Limitations of the study

This case report has several limitations. First, the follow-up period available at the time of manuscript preparation was relatively short, limiting the ability to assess long-term recurrence. Second, the findings reflect a single-center, single-surgeon experience, which may reduce generalizability. Third, the absence of a control group or comparative cohort restricts the capacity to draw broader conclusions regarding the superiority of this minimally invasive approach. Selection bias is also inherent, as the patient was deemed a suitable candidate for a totally endoscopic strategy. Additionally, a formal cost-effectiveness analysis was not performed. Given these limitations, further research involving larger, multi-center studies is necessary to validate the reproducibility, long-term durability, and broader applicability of this technique.

## Conclusion

Totally endoscopic redo surgery for recurrent left atrial myxoma is a feasible and safe approach, even in the presence of adhesions. The right atrial access, DCT approach, and partial interatrial septal resection optimize tumor removal while minimizing recurrence risk. Our findings support this minimally invasive strategy as an effective alternative in carefully selected redo cases, ensuring surgical precision and favorable outcomes.

Full Surgical Video link:

https://drive.google.com/file/d/1kyca-FD8Cio_SlUmsHDajlbAnKQCpOxH/view?usp= sharing

## Declarations

**IRB approval:** UMC Institutional Policy dictates that single patient reports do not require institutional.

**Informed consent statement:** Written informed consent was obtained from the patient for publication of this case report and any accompanying images. A copy of the written consent is available for review by the Editor-in-Chief of this journal.

**Funding:** No funding was received for this study.

**Availability of data and materials:** All of the material is available and owned by the authors and/or no permissions are required.

## Author contribution statement

Surgery: PTVC, PQT, LDH. Writing: PQT. Revision: PQT and NHD, Assistant: CDK and LDH.

## References

[1] Tolu-Akinnawo O, Dufera RR, Ramanna N (2023). Recurrent left atrial myxoma: The significance of active surveillance. Cureus.

[2] Thuan PQ, Chuong PTV, Dinh NH (2023). Adoption of minimally invasive mitral valve surgery: single-centre implementation experience in Vietnam. Ann Med Surg (Lond).

[3] Pollari F, Weber L, Fischlein TJJoVS (2021). Giant myxoma removal through a 3D-4K minimally invasive thoracoscopy: a case report and step-by-step guide.

[4] Viet Chuong PT, Thuan PQ, Thang HD, Huy LD, Bao Luan TM, Dinh NH Totally endoscopic replacement of the ascending aorta with 3-dimensional visualization: Defining totally endoscopic? A case report. JTCVS Techniques.

[5] Bjessmo S, Ivert T (1997). Cardiac myxoma: 40 years’ experience in 63 patients. Ann Thorac Surg.

[6] Keeling IM, Oberwalder P, Anelli-Monti M, Schuchlenz H, Demel U, Tilz GP, Rehak P, Rigler B (2002). Cardiac myxomas: 24 years of experience in 49 patients. Eur J Cardiothorac Surg.

[7] Hermans K, Jaarsma W, Plokker HW, Cramer MJ, Morshuis WJ (2003). Four cardiac myxomas diagnosed three times in one patient. Eur J Echocardiogr.

[8] Rathore KS, Hussenbocus S, Stuklis R, Edwards J (2008). Novel strategies for recurrent cardiac myxoma. The Annals of Thoracic Surgery.

[9] Mahavar RK, Arora D, Singh A, Mishra M (2021). Recurrent cardiac myxoma: A case report. Ann Card Anaesth.

[10] Bireta C, Popov AF, Schotola H, Trethowan B, Friedrich M, El-Mehsen M, Schoendube FA, Tirilomis T (2011). Carney-complex: Multiple resections of recurrent cardiac myxoma. Journal of Cardiothoracic Surgery.

[11] Mujtaba SS, Clark SC (2018). Extended trans-septal *versus* left atrial approach in mitral valve surgery: 1017 patients’ experience. Heart Asia.

[12] Stratakis CA, Kirschner LS, Carney JA (2001). Clinical and molecular features of the Carney complex: diagnostic criteria and recommendations for patient evaluation. J Clin Endocrinol Metab.

[13] Jang MY, Lee JH, Heo M, Lim SK, Chung SR, Sung K, Kim WS, Cho YH (2023). Impact of interatrial septal reconstruction on atrial tachyarrhythmia after surgical resection of myxoma. J Chest Surg.

[14] Ekinci EI, Donnan GA (2004). Neurological manifestations of cardiac myxoma: a review of the literature and report of cases. Intern Med J.

[15] Perchinsky MJ, Lichtenstein SV, Tyers GF (1997). Primary cardiac tumors: forty years’ experience with 71 patients. Cancer.

